# Chemical engineering of zein with polyethylene glycol and Angiopep-2 to manufacture a brain-targeted docetaxel nanomedicine for glioblastoma treatment

**DOI:** 10.1007/s13346-024-01659-x

**Published:** 2024-07-15

**Authors:** Seem Awad, Marco Araújo, Paulo Faria, Bruno Sarmento, Cláudia Martins

**Affiliations:** 1 i3S– Instituto de Investigação e Inovação em Saúde, Universidade do Porto, Rua Alfredo Allen 208, Porto, 4200-135 Portugal; 2 INEB– Instituto de Engenharia Biomédica, Universidade do Porto, Rua Alfredo Allen 208, Porto, 4200- 135 Portugal; 3 IUCS-CESPU - Instituto Universitário de Ciências da Saúde, Gandra, 4585-116 Portugal

**Keywords:** Angiopep-2, Blood-brain barrier, Docetaxel, Drug delivery, Glioblastoma, Nanoparticles, Targeted nanomedicine, Zein

## Abstract

Glioblastoma (GBM) is the deadliest adult brain cancer. The current standard-of-care chemotherapy using orally administered temozolomide (TMZ) presents poor improvement in patient survival, emphasizing the compelling need for new therapies. A possible chemotherapeutic alternative is docetaxel (DTX), which possesses higher tumoricidal potency against GBM cells. However, its limited blood-brain barrier (BBB) permeability poses a constraint on its application. Nonetheless, nanomedicine offers promising avenues for overcoming this challenge. Angiopep-2 (ANG2) is a peptide that targets the BBB-overexpressed low-density lipoprotein receptor (LDLR). In this work, we managed, for the first time, to employ a pioneering approach of covalently linking zein protein with polyethylene glycol (PEG) and ANG2 prior to its formulation into nanoparticles (ZNPs) with enhanced stability and LDLR-mediated brain targetability, respectively. Carbodiimide and click chemistry approaches were optimized, resulting in functional modification of zein with around 25% PEG, followed by functional modification of PEG with nearly 100% ANG2. DTX-loaded ZNPs presented 100 nm average size, indicating high suitability for BBB crossing through receptor-mediated transcytosis. ZNPs maintained the cytotoxic effect of the loaded DTX against GBM cells, while demonstrating a safe matrix against BBB cells. Importantly, these brain-targeted ZNPs showcased up to fourfold enhancement in blood-to-brain permeability in a BBB in vitro model, highlighting the potential of this novel approach of BBB targeting in significantly improving therapeutic outcomes for GBM patients. The versatility of the system and the possibility of significantly increasing drug concentration in the brain open the door to its future application in a wide range of other brain-related diseases.

**Graphical abstract:**

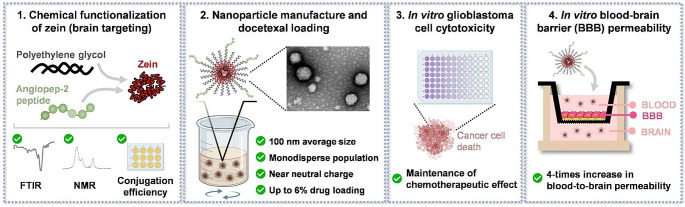

**Supplementary Information:**

The online version contains supplementary material available at 10.1007/s13346-024-01659-x.

## Introduction

Glioblastoma (GBM) is an extremely aggressive malignant brain cancer that has been classified as a grade IV brain tumour by the World Health Organization, thereof denoting one of the most lethal and devastating human cancers to ever exist [1]. Characterized by its extremely poor prognosis and its short 15-month median overall survival rate [2], GBM possesses high recurrence rates reaching over 90% [3]. With GBM being the most prevalent type of brain cancer in adults, it continues to pose scientists with stagnant challenges for finding efficacious and patient-oriented therapies beyond end-of-life palliative care.

The current standard-of-care therapy for GBM is the Stupp Protocol which involves maximal surgical resection of the tumour, followed by cycles of chemotherapy with the DNA alkylating agent temozolomide (TMZ, oral administration) alongside concomitant radiotherapy, later followed by adjuvant doses of TMZ for a period of six months [4]. However, beyond TMZ’s high aqueous solubility (5.09 mg/mL) [5] and relatively good blood-brain barrier (BBB) permeability (around 30-40% blood dose) [6], its limited tumoricidal potency (half maximal inhibitory concentration (IC50) of 1–2 mM against U-87 MG GBM cell line) [7] and mechanisms of tumour resistance [8] underscore the pressing need for novel therapeutic interventions. Amongst potential alternatives studied is the microtubule inhibitor docetaxel (DTX), with a significantly higher potency against GBM cells owing to its 100,000 times lower IC_50_ compared to TMZ [7]. However, DTX’s suboptimal aqueous solubility and highly limited BBB permeability (< 1% blood dose) [9] pose a constraint in using the drug in its native form to fight GBM, as this leads to insufficient accumulation in the brain parenchyma and considerable off-target biodistribution. Consequently, altering the drug delivery mechanism of DTX and improving its pharmacokinetic properties could be a key aspect in circumventing the disadvantages imposed by it as a treatment for GBM.

Drug delivery through nanomedicine showcases a versatile platform that underlines essential routes to mitigate the pharmacokinetic problems of DTX. This is due to their versatile structure and stealth properties, which present an opportunity for an efficient transport across biological barriers, enhanced drug solubilization, reduced side effects, and improved tissue targeting and accumulation [10]. With that being said, the major hurdle associated with drug delivery to the brain lies within the highly selective BBB which acts as a protective barrier against the entry of foreign substances. This is due to the presence of an extensive network of tight junctions which results in low rates of transcytosis and restricted paracellular permeability [11]. Resultantly, to address this issue, the concept of receptor-mediated transcytosis has been thoroughly explored. This ‘trojan horse’ concept of targeted nanomedicines involves the exploitation of specific naturally occurring transporters found across the BBB to facilitate the transport of therapeutics into the brain, bypassing this critical biological barrier [12]. In the case of GBM, a highly expressed receptor in the BBB endothelium is the low-density lipoprotein-receptor (LDLR). LDLR is an ApoE receptor that plays a key role in maintaining vascular endothelial homeostasis through the shuttling of cholesterol [13]. Binding to LDLR initiates clathrin or caveolin-mediated endocytosis, eventually culminating in the transcytosis of the engulfed molecules across the BBB [12, 14]. A thoroughly studied peptide targeting the LDLR and currently being used in cancer nanomedicine is Angiopep-2 (ANG2) [15]. ANG2 is a 19-amino acid synthetic peptide with a sequence of TFFYGGSRGKRNNFKTEEY which is derived from the Kunitz domain of the protein aprotinin and that had been constructed to selectively interact with LDLR [16].

In the pursuit of effective nanocarriers for encapsulating the highly hydrophobic drug DTX, polymeric zein nanoparticles (ZNPs) have attracted attention in the field of drug delivery for employing a natural protein derived from corn seeds, offering the advantages of high biodegradability and inexpensiveness [17]. Zein is also considered a Generally Regarded as Safe ‘GRAS’ ingredient by the FDA where its multifunctional properties extend beyond the realm of pharmaceutical applications, with previous use as an excipient in food production [18]. Additionally, zein’s amphiphilic nature, which allows it to be formulated in the absence of surfactant molecules [19], offers an exciting opportunity to be exploited in drug delivery. Ergo, leveraging the unique properties of this naturally occurring protein into intravenous drug delivery of highly hydrophobic drugs such as DTX. Polyethylene glycol (PEG) is often use to modify the ZNPs surface, increasing the stability of their highly hydrophobic core and circulation time.

Here, we propose for the first time the chemical modification of zein with PEG and ANG2 to manufacture a DTX nanomedicine for GBM treatment with brain targeting properties. The chemical design was carefully optimized, followed by ZNPs assembly and physicochemical characterization. The nanomedicine was evaluated regarding cytotoxic potential against GBM cells, and blood-to-brain permeability was screened in a BBB in vitro model.

## Materials and methods

### Materials

Zein, resazurin, trypsin-EDTA, human basic fibroblast growth factor (bFGF), hydrocortisone, N-dicyclohexylcarbodiimide hydrochloride (DCC), N-hydroxysuccinamide (NHS), N,N-Diisopropylethylamine (DIPEA), tris(2-carboxyethyl)phosphine hydrochloride (TCEP), coumarin-6 (C6), N, N-Dimethylformamide (DMF), sodium chloride, Ellman’s reagent (5,5′-dithiobis-2-nitrobenzoic acid), uranyl acetate, sodium phosphate dibasic heptahydrate, and sodium phosphate monobasic monohydrate were purchased from Sigma-Aldrich (Massachusetts, US). Maleimide-PEG-Carboxylic acid (5 kDa PEG, Mal-PEG5k-COOH) was bought from RuixiBiotech^®^ (Jiangsu, CN). Thermo Fisher Scientific^®^ (Massachusetts, US) provided acetonitrile (ACN), diethyl ether, methanol, dimethyl sulfoxide (DMSO), Dulbecco’s Minimum Essential Medium (DMEM) high glucose with pyruvate, Hank’s Balanced Salt Solution (HBSS), non-essential amino acids 100x, triton-X-100, rat tail collagen type I, CD lipid concentrate and penicillin-streptomycin (P/S). Endothelial basal medium (EBM-2) was provided by Lonza^®^ (Basel, CH). Heat-inactivated fetal bovine serum (HI-FBS) was supplied by LabClinics^®^ (Barcelona, ES). Docetaxel was bought from LC Labs^®^ (Massachusetts, US). Cysteine-modified ANG2 was supplied by GenScript (NH2-CTFFYGGSRGKRNNFKTEEY-COOH, New Jersey, US). The human brain microvascular endothelial cell line (hCMEC/D3) and the human Uppsala 87 malignant GBM cell line (U-87 MG) were both purchased from American Type Culture Collection (Virginia, US).

### Chemical modification of zein

#### Covalent PEGylation

Carbodiimide-mediated coupling was employed to conjugate Mal-PEG5k-COOH with the glutamine moieties of the zein protein, giving PEGylated zein (zein-PEG5k-Mal). Six different conditions with varying molar ratios of zein, PEG, DCC and NHS and/or time of the reaction were attempted to obtain these conjugates of zein and PEG (ZP), as summarized in Table 1. A vial containing 200 mg of zein dissolved in 5 mL anhydrous DMF was placed on a magnetic stirrer to stir for 2 h under inert conditions. DIPEA (248 µL, 0.355 mmol) was also added to the vial, acting as a proton scavenger for the subsequent amide coupling reaction. In another vial, Mal-PEG5k-COOH, DCC and NHS were dissolved in 4 mL anhydrous DMF and were left to react for 2 h stirring under inert conditions. Afterwards, the content of the Mal-PEG5k-COOH vial was transferred to the zein vial and left stirring on ice for 10 min, followed by stirring at room temperature (reaction times in Table 1). The reactions were qualitatively followed by thin layer chromatography (TLC) using bromocresol staining to follow complete activation of the Mal-PEG5k-COOH carboxylic acid (Figure S1). Finally, the samples were lyophilized overnight in a -80 °C freeze dryer set for 0.014 mbar (LabConco, US) to obtain the solid conjugates, which were stored at − 20 °C until further use.

**Table 1:** Summary of the experimental conditions tested to produce zein-PEG5k-Mal conjugates

Product code	Molar ratio zein: Mal-PEG5k-COOH: DCC: NHS	Reaction time (h)
*ZP1*	1:2:2:2	24
*ZP2*	1:4:4:4	24
*ZP3*	1:4:4:4	48
*ZP4*	1:4:40:100	24
*ZP5*	1:2:20:50	24
*ZP6*	1:4:40:100	24

#### PEG functionalization with ANG2

The linkage of cysteine-modified ANG2 to zein-PEG5k-Mal was performed using a thiol-maleimide click chemistry reaction under inert atmosphere (0.6:1 molar ratio of cysteine-modified ANG2: zein-PEG5k-Mal, respectively), adapting a procedure reported by *Martins et al.*. [20]. In brief, cysteine-modified ANG2 (2.8 mg, 1.164 µmol) were mixed with 0.8 mg TCEP (molar ratio of 1:2 of cysteine-modified ANG2:TCEP, respectively) in 1.5 mL of anhydrous DMF and stirred for 1 h at RT. 50 mg of zein-PEG5k-Mal were dissolved in 1 mL of anhydrous DMF, then added to the previous ANG2 solution and kept reacting for 24 h at 4 °C under stirring. The product, a conjugate of ZP and ANG2 (ZP-ANG2), was then precipitated in 25 mL cold diethyl ether and centrifuged at 4 °C, 3220 *g* for 20 min. The resultant pellet was centrifuged twice with 5 mL of Milli-Q water at 4 °C and 3220 *g* for 10 min. The product and supernatants were freeze-dried overnight (-80 °C freeze dryer set for 0.014 mbar) and stored to assess quantification of the grafted peptide through Ellman’s assay.

**Fig. 1:**
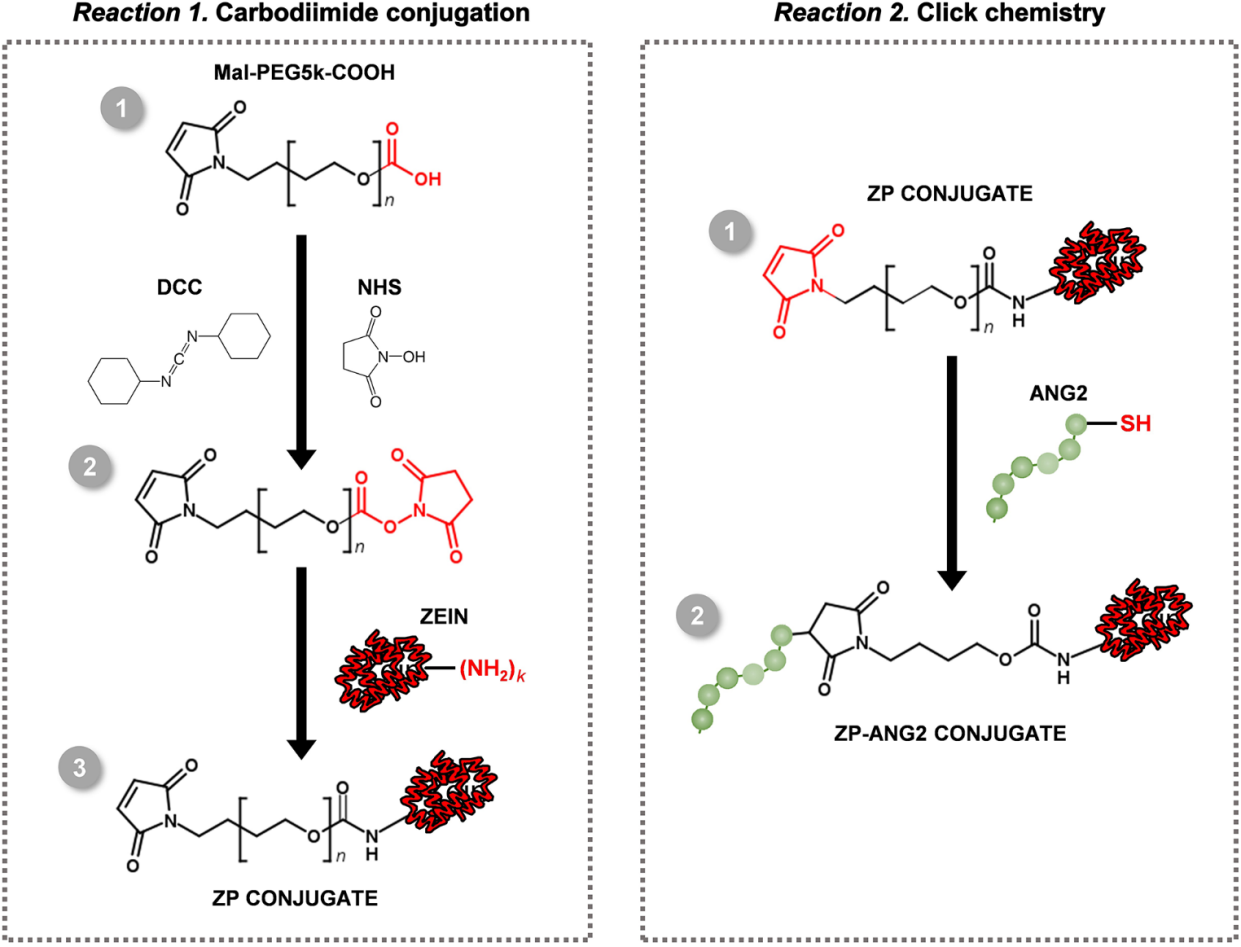
Scheme showing the reactions performed to obtain the PEGylated and ANG2-functionalized zein conjugates. Red color indicates the chemical groups involved in each step of reaction

The chemical reactions taking place during the PEGylation and subsequent ANG2 functionalization of the zein protein are summarized in Fig. 1.

### Characterization of the zein chemical conjugates

#### Attenuated total reflectance–fourier transform infrared spectroscopy (ATR-FTIR)

The ATR-FTIR spectra of the raw materials and conjugates were obtained using a Fourier transform spectrophotometer IR Affinity-1 S (Shimadzu, JP) coupled to a Golden Gate Attenuated Total Reflectance. 2 mg of the raw material and freeze-dried samples were tested, and the reflectance spectra were obtained by scanning from 400 to 4000 cm^− 1^ at 4 cm^− 1^ resolution, using 32 scans per spectrum. The spectra were analyzed using Spectragryph^®^ 1.2 software.

#### ^1^H nuclear magnetic resonance (NMR)

5 mg of the raw material and freeze-dried samples were dissolved in 600 µL of a mixture of deuterated methanol and deuterated water (75:25 (v/v) ratio, respectively) and analyzed at 25 °C using a Bruker Advance III 400 MHz (Bruker Corporation, US). The data obtained were analyzed using MestReNova^®^ 6.0.2 software.

#### ANG2 conjugation efficiency

The supernatants of the coupling reaction of cysteine-modified ANG2 to zein-PEG5k-Mal (Sect. 2.2.2) were freeze-dried and resuspended in 1 mL of Milli-Q^®^ water to quantify the unbound cysteine-ANG2. 50 µL of 4 mg/mL Ellman’s reagent were mixed with 2.5 mL of sodium phosphate reaction buffer (pH 8.0). 250 µL of the sample was later added and left to react for 15 min at RTP. The absorbance was read at 412 nm using a Synergy MX plate reader (BioTek, US). The amount of unbound ANG2 was given by the difference between the initial amount of the peptide and the amount of the peptide detected in the washing supernatants. The samples were evaluated in triplicate.

### Nanoparticle manufacture and characterization

#### Production of ZNPs

To prepare ZNPs, a previously reported nanoprecipitation method was modified [17, 21]. Briefly, zein or its conjugates were dissolved in 70% (v/v) ethanol solution at a concentration of 5 mg/mL to produce the nanoparticles (testing conditions summarized in Table 2). ZNPs were loaded with 8% DTX (theoretical loading) dissolved in 80 µL of DMSO, and directly added to the initial organic phase. The solution was left to stir at 500 rpm and RT for 1 h, to ensure complete zein dissolution, before inducing nanoprecipitation by the addition of an equivalent volume of Milli-Q^®^ water under continuous stirring at 400 rpm and RT for 3–4 h. Then, 250 µL of ZNPs were diluted with 250 µL of Milli-Q^®^ water and washed using a 100 kDa molecular weight cutoff 0.5 mL Amicon^®^ ultrafilter device (Millipore Corporation, US) at 14,000 g and RT, until a retentate volume of 250 µL was reached. Blank ZNPs were used as controls and were prepared using the same protocol but without DTX. Fluorescent ZNPs were formulated similarly, using 0.1% (w/w) C6 as described in the literature [22].

**Table 2:** Relative composition of the different zein nanoformulations, indicating the equivalent percentage of PEGylation and ANG2

Formulation code	Percentage of component in the formulation (%)			Equivalent percentage of PEGylation (%)	Equivalent percentage of ANG2 (%)
	Zein	ZP4	ZP4-ANG2		
NF ZNPs	100	0	0	0	0%
ZP4 ZNPs	20	80	0	20	0%
ZP4-ANG2 5% ZNPs	20	60	20	20	5%
ZP4-ANG2 10% ZNPs	20	40	40	20	10%
ZP4-ANG2 20% ZNPs	20	0	80	20	20%

#### Hydrodynamic diameter, polydispersity index, and zeta-potential

The hydrodynamic diameter, polydispersity index (PDI) and zeta-potential and were measured using dynamic light scattering (DLS) coupled with laser Doppler electrophoresis by means of a Zetasizer^®^ Nano ZS (Malvern, UK). The nanoparticles were diluted at a ratio of 1:100 with 10 mM NaCl adjusted prior to use to pH 4.5. Three measurements were taken for each run.

#### Morphological evaluation

The non-PEGylated, PEGylated, and ANG2 functionalized ZNPs’ morphologies were confirmed by transmission electron microscopy (TEM). TEM images were obtained by mounting 5 µL of the NP suspension stained with 1% uranyl acetate on carbon film-coated mesh nickel grids (Electron Microscopy Sciences, US). Visualization was carried out under a 120 kV TEM (JEOL-JEM 1400, JP). Images were digitally recorded using a CCD camera (Orious 1100 W, JP).

#### Association efficiency (AE) and drug loading (DL)

The amount of loaded DTX was directly quantified by high-performance liquid chromatography (HPLC) in order to determine the association efficiency (AE) and loading degree (LD) of the developed formulations by dissolving the nanoparticles in DMF. The quantification of DTX was performed using a LaChrom^®^ HPLC system (Merck, US) with a Symmetry reverse-phase C18 column (5 μm, 125 × 4 mm; Milford, US) and a LiChrospher 100 RP-18 guard column (Merck, US). The HPLC method employed a gradient elution with a flow rate of 1 mL/min. The mobile phase composition varied as follows: 10:90 ACN: H_2_O from 0 to 5 min, 60:40 ACN: H_2_O from 5 to 14 min, and 10:90 ACN: H_2_O from 14 to 17 min. The drug was retained at around 9.60 min. 50 µL of each sample was injected and the detection was performed at 227 nm using a UV detector [23]. The AE (%) was determined as a percentage of the experimental drug payload to the theoretical drug payload. DL (%) was calculated as a percentage of the quantified DTX mass to the total mass of the nanoparticles.

### Cell culture

hCMEC/D3 cells were cultured in EBM-2 medium (supplemented with 5% (v/v) FBS, 1% (v/v) P/S, 0.05% (v/v) hydrocortisone, 0.5% (v/v) ascorbic acid, 1% (v/v) CD lipid concentrate and 1% (v/v) HEPES buffer). 200 ng/mL bFGF was added extemporaneously in the culture medium. The U-87 MG cells were cultured in high glucose with pyruvate DMEM supplemented with 10% (v/v) FBS and 1% (v/v) P/S. All the procedures involving the culturing of these cell lines were performed under BioAir Class II BSC Biological Safety Cabinet– Airstream^®^ (Portland, US) with a vertical HEPA filtering system.

### Evaluation of cell metabolic activity

U-87 MG cells or hCMEC/D3 were seeded individually into 96-well plates with a density of 1 × 10^4^ and 0.8 × 10^4^ cells per well, respectively. The following day, cells were treated with different concentrations of blank and/or loaded nanoparticles. A positive control (1% (v/v) of Triton X-100; 0% metabolic activity), and a negative control (supplemented media only; 100% metabolic activity) were prepared, and the plates were incubated for 24 h, 48–72 h at 37 °C. The media was then replaced with 20% (v/v) of media-diluted resazurin, and the plates were incubated at 37 °C for 3 h in the dark. Fluorescence was measured at an excitation and emission wavelengths of 530 and 590 nm, respectively, using the microplate reader. Experiments were performed at least in triplicate, and all data were normalized regarding the negative and positive controls. Graphical data for cell metabolic activity was referenced to a threshold of 70% cell viability according to the ISO 10993-5 standard [24].

### Assessment of in vitro BBB permeability

For setting up the BBB in vitro model, previously optimized protocols were followed [24, 25]. The membrane of a PET clear insert system of 1.0 μm pore size (cellQART^®^, DE) was coated with 90 µL rat tail collagen type I (50 µg/mL solution) for 1 h, and then washed twice with PBS. hCMEC/D3 cells were cultured at a density of 2.5 × 10^4^ cells/cm^2^ in 0.5 mL of media on the apical side of the insert. 1.5 mL of EBM-2 media were placed in the basolateral compartment of each insert, and the media was replaced every two days. A blank insert was prepared in the same manner, but without seeding cells. Cells were cultured for 8 days at 37 °C in a 5% CO_2_ incubator. Cell monolayer integrity was periodically analyzed by determining the TEER using an endothelial voltohmmeter (World Precision Instruments, US). The resistance (Ω.cm^2^) of a blank filter was subtracted from each measurement (Eq. 1).

1 $$\eqalign{{\rm{TEER}}\,{\rm{(}}\Omega {\rm{.c}}{{\rm{m}}^{\rm{2}}}{\rm{) }}\, & {\rm{ = }}\,\left( {{\rm{TEE}}{{\rm{R}}_{{\rm{seeded\,insert}}}}\, - \,{\rm{TEE}}{{\rm{R}}_{{\rm{blank\,insert}}}}} \right) \cr & {\rm{ \times }}\,{\rm{Insert }}\,{\rm{Area }}\,{\rm{(c}}{{\rm{m}}^{\rm{2}}}{\rm{)}} \cr} $$

At day 8, the permeability of fluorescently labeled nanoparticles was conducted at 37 ^º^C using an orbital shaker set at 100 rpm. At each time point (4, 8, 24 h), 100 µL samples were withdrawn from the basolateral side, and the fluorescence was measured against a calibration curve at 456/500 nm (excitation/emission wavelength) to determine the mass of nanoparticles that crossed the BBB monolayer. The permeability percentage across the cell monolayer was calculated from the masses measured in the basolateral compartment at each timepoint, as a fraction of the initial apical mass. Whereas, the apparent permeability coefficient (P_app_) at 24 h was calculated using Eq. 2. The experiments were performed in triplicate.

2 $${{\rm{P}}_{{\rm{app}}}}\,{\rm{ = }}\,\left( {{{\Delta {\rm{Q}}} \over {\Delta {\rm{t}}}}} \right)\,{\rm{ \times }}\,\left( {{{\rm{1}} \over {{\rm{A }}{{\rm{C}}_{\rm{0}}}}}} \right)$$

where ΔQ/Δt is the steady-state flux (µg/s), C_0_ is the initial concentration of DTX in the apical compartment (µg/mL), and A is the insert area (cm^2^).

### Statistical analysis

Results are represented as mean ± standard deviation (STD) from a minimum of three experiments. To analyze the data, 2-tailed paired Student’s t-test, or two-way analysis of variance (ANOVA) followed by a post hoc test (Dunnett’s test), were used. Significance levels were denoted as *p < 0.05, **p < 0.01, or ***p < 0.001. Statistical calculations were carried out using GraphPad Prism 9 software by GraphPad Software, USA.

## Results and discussion

### Covalent PEGylation of zein

Zein’s biocompatibility, biodegradability, and ability to effectively encapsulate drugs make it an ideal matrix for drug delivery systems. However, the disadvantages of zein, such as its rapid clearance, limited solubility, and potential immunogenicity, necessitate PEGylation to overcome these challenges, hence ensuring safe and efficient delivery of therapeutic agents.

Here, we propose the covalent PEGylation of zein through its carbodiimide coupling with Mal-PEG5k-COOH. Based on reports in the literature, glutamine and asparagine account for 22% of the zein structure [26]. For that reason, the carboxylic acid group of Mal-PEG5k-COOH was used for the carbodiimide conjugation to target the glutamine and asparagine’s primary amines of zein. The amino acid cysteine in zein’s structure, on the other hand, accounts for less than 1% of the zein structure [27], reducing the probability of the Michael-type addition cross-reactivity between the thiol chemical group of cysteine and the maleimide moiety found on Mal-PEG5k-COOH.

**Fig. 2:**
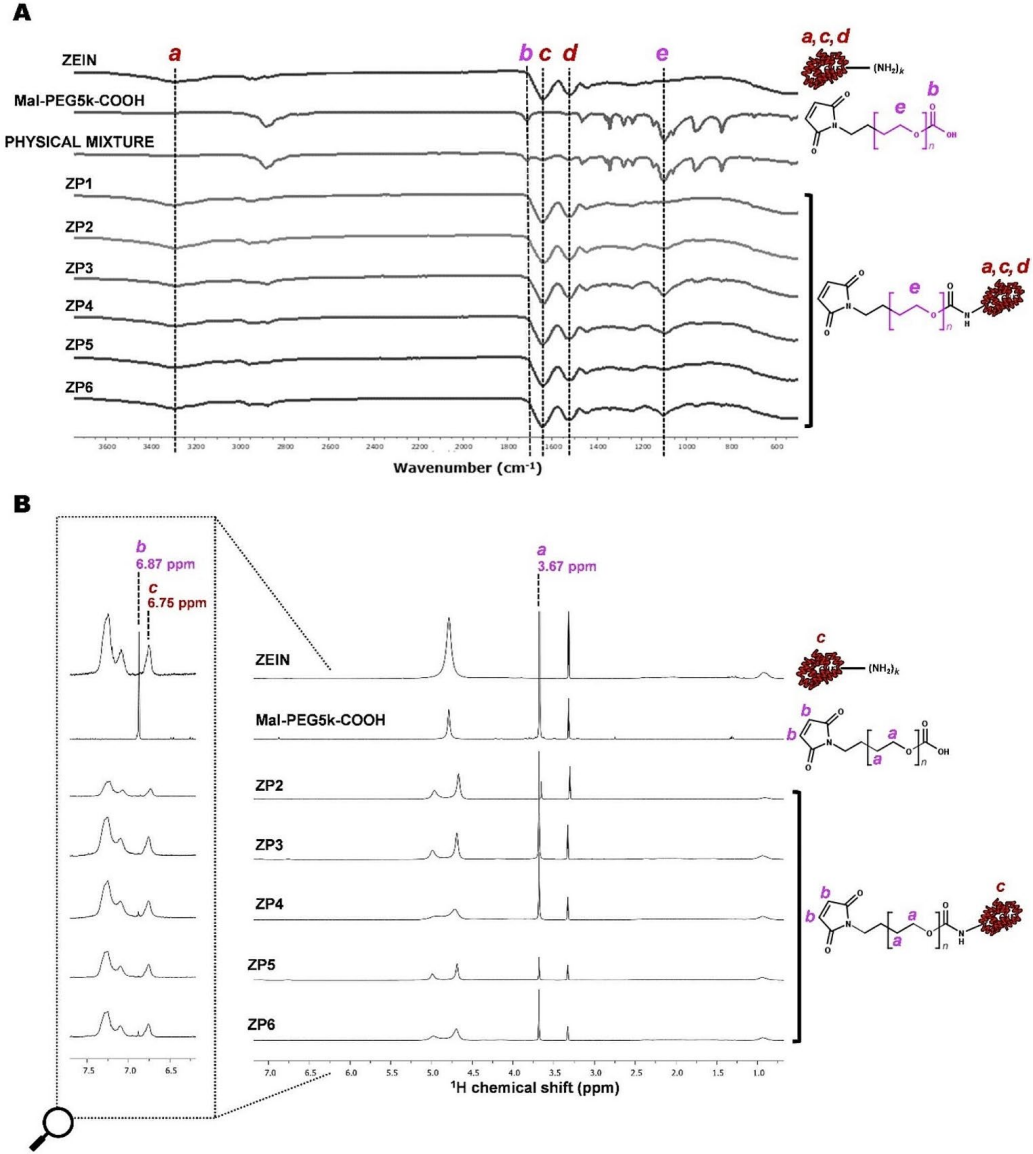
**(A)** ATR-FTIR and **(B)**^1^H-NMR spectra of zein, Mal-PEG5k-COOH, physical mixture of zein and Mal-PEG5k-COOH, or ZP conjugates. **(A)***a*, *c* and *d* represent the major distinctive bands of zein used for following the conjugation reaction, while *b* and *e* are characteristic bands of Mal-PEG5k-COOH. **(B)***a* and *b* represent peaks characteristic of Mal-PEG5k-COOH, while *c* represents a peak characteristic of zein. Peaks *a* and *c* were used to calculate the conjugation efficiency of Mal-PEG5k-COOH to zein

To verify the success of the zein PEGylation reaction, the ATR-FTIR spectra of zein-PEG5k-Mal (ZP1-ZP6 conjugates), zein, Mal-PEG5k-COOH and stoichiometric control physical mixtures (zein and Mal-PEG5k-COOH) were analyzed. Generally, the ATR-FTIR spectrum of zein showed -NH_2_ stretching bands present at around 3300 cm^− 1^ which were typical for the unconjugated zein (*band a*), along with the characteristic bands of the zein protein backbone of C = O of amide I stretching and amide II N-H bending coupled to -C-N stretching at 1643 cm^− 1^ (*band c*) and 1542 cm^− 1^ (*band d*, respectively [28]. Alternatively, Mal-PEG5k-COOH spectra showed an imminent PEG-related band at around 1100 cm^− 1^ (*band e*), along a band at around 1700 cm^− 1^ corresponding to the C = O stretching vibration of carboxylic acid groups (band *b*). Zein bands (*a, c*, *d*) were found in the spectra of all zein-PEG5k-Mal conjugates. However, no new bands were observed in the spectra of the conjugates as the new amide bonds formed overlap with the protein amide peaks [29]. The success of the PEGylation reaction was confirmed both by the disappearance of the band characteristic of the carboxylic acid stretching vibrations of Mal-PEG5k-COOH (band *b*), and the increase of its band characteristic of the PEG spectra (band *e*). This band characteristic of the PEG spectra was, however, absent in the ZP1 spectrum, but present in the spectra of conjugates ZP2 to ZP6 (Fig. 2A). This undetectable amount of conjugated PEG in ZP1, which suggests the failure of an efficient conjugation, could be a consequence of the low amount of Mal-PEG5k-COOH used in the reaction, as reports have shown that the key factor affecting the conjugation efficiency is the ratio of carboxylic acid functionalities (-COOH) to the amine (-NH_2_) groups of the protein [30], where an excess of Mal-PEG5k-COOH is necessary to prevent intermolecular crosslinking within the protein [31].

ZP2-ZP6 were then analyzed by NMR to evaluate the conjugation efficiency of Mal-PEG5k-COOH to zein, determining the degree of PEGylation and amount of available maleimide groups for subsequent reaction with ANG2. ^1^H peak of ethylene glycol fragment from PEG (-CH_2_-CH_2_) were present at around δ = 3.67 ppm as shown in Fig. 2B (peak *a*) [32]. Whereas, maleimide peak in the Mal-PEG5k-COOH was found at around δ = 6.87 ppm (peak *b*) (Fig. 2B). Regarding zein, the protons from its aromatic amino acid fragments at around δ = 6.75 ppm (peak *c*) were considered (Fig. 2B). Peak *c* denotes two protons corresponding to the aromatic ring from tyrosine. By comparing the zein representative peaks (tyrosine) to Mal-PEG5k-COOH representative peaks (ethylene glycol) integrals, the percentage of conjugation of zein to Mal-PEG5k-COOH was calculated for each of the conjugates (Table 3).

**Table 3:** Percentage of conjugation of zein to Mal-PEG5k-COOH for each ZP conjugate

Product code	Mal-PEG5k-COOH conjugation efficiency (%)
*ZP1*	*Undetectable*
*ZP2*	16.7
*ZP3*	28.5
*ZP4*	26.5
*ZP5*	21.2
*ZP6*	21.9

Considering the good balance between PEGylation efficiency value and a reaction time of only 24 h, ZP4 was selected to proceed with further studies.

### ANG2 functionalization of PEGylated zein

ANG2 peptide offers enhanced brain targeted drug delivery by efficiently shuttling drug delivery systems across the BBB, facilitating the accumulation of therapeutic agents in the brain parenchyma and, consequently, improving treatment efficacy. As such, here, we propose the modification of covalently PEGylated zein with ANG2 to enable an efficient brain targeted drug delivery, harnessing the advantages of both technologies for improved therapeutic outcomes in central nervous system disorders. Thiol-maleimide click chemistry was used to conjugate the maleimide chemical group of zein-PEG5k-Mal (ZP4) with the thiol chemical group of a cysteine-modified ANG2.

The efficiency of ZP4 chemical conjugation with ANG2 (giving ZP4-ANG2) was indirectly assessed by quantification of the free thiol chemical groups of the peptide, projecting on the unbound cysteine-modified ANG2 present in the supernatant collected from the conjugates’ washings. This quantification was performed using the Ellman’s assay. The conjugation efficiency of ANG2 was calculated as a function of the initial peptide mass and was found to be 96.43 ± 0.30%. Thus, this conjugation process demonstrated high grafting efficiency of ANG2 to the PEGylated zein. This implies that for every maleimide chemical group present in zein-PEG5k-Mal, a corresponding ANG2 moiety was successfully conjugated.

The percentage of ANG2 in the ZP4-ANG2 conjugate was also retrieved from the NMR spectra obtained for ZP4 (Fig. 2B). The percentage of covalently PEGylated zein in ZP4 was rounded down to 25% (Table 3), obtained as a function of the percentage of maleimide chemical groups on the conjugated Mal-PEG5k-COOH. Considering from the Ellman’s assay that most of these maleimide moieties on ZP4 were occupied by ANG2, the peptide covered approximately 25% of the zein protein surface.

### Production and characterization of ZNPs formulations

Several ZNP nanoformulations loaded with DTX were produced through a straightforward nanoprecipitation process *a posteriori* from a successful covalent PEGylation and ANG2 modification of zein. ZNP formulations were produced with 5%, 10% or 20% ZP4-ANG2 (ZP4-ANG2 5%, ZP4-ANG2 10% and ZP4-ANG2 20% ZNPs, respectively). Control nanoformulations without ANG2 were also produced, based on covalently PEGylated zein (ZP4 ZNPs) or only pure zein (NF ZNPs).

**Fig. 3:**
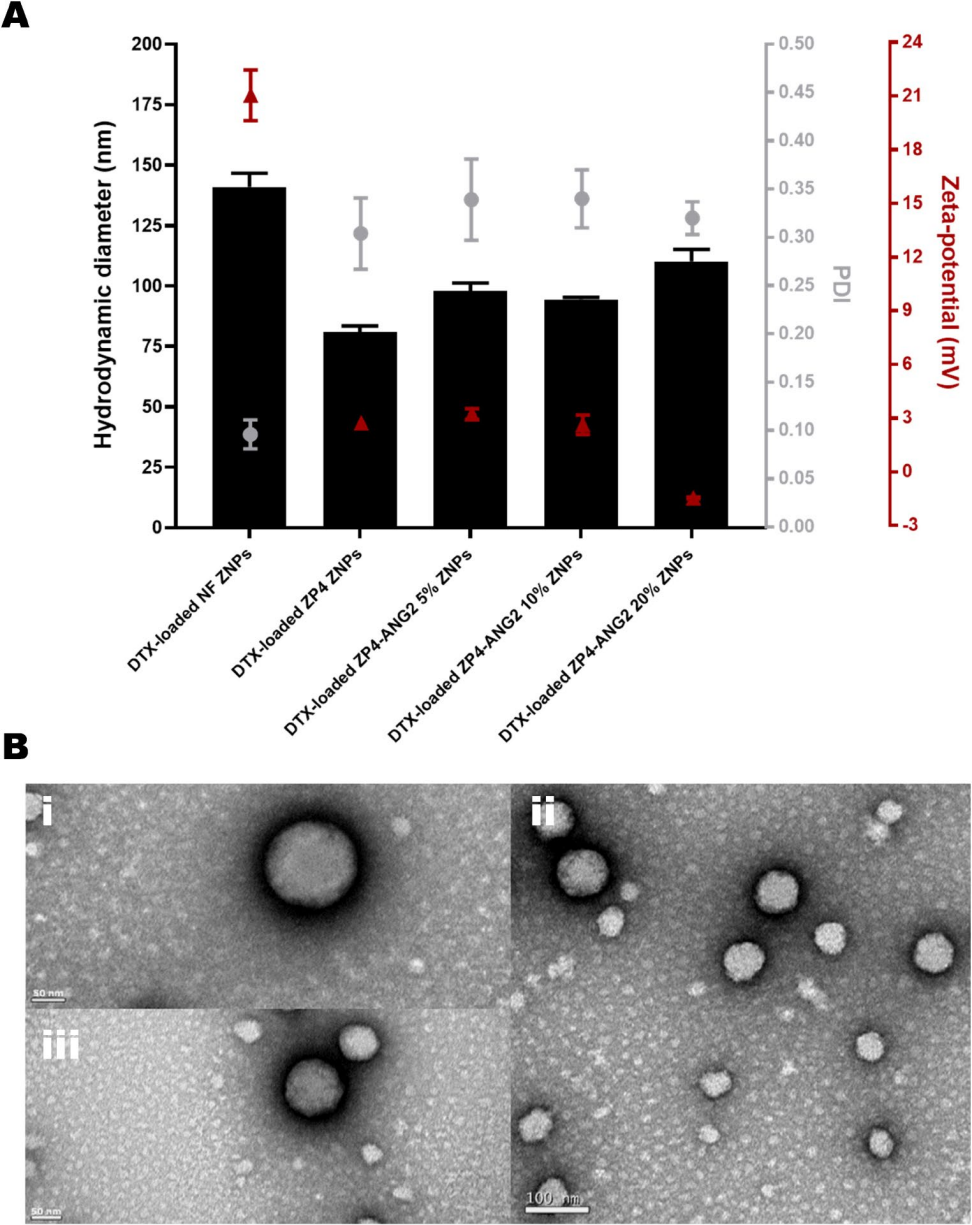
**(A)** Summary of average hydrodynamic diameter, PDI and zeta-potential values for the different nanoformulations of zein loaded with DTX. Data presented as mean ± STD (*n* = 3, each *n* corresponding to a different nanoformulation batch). **(B)** TEM images of (i) DTX-loaded NF ZNPs, (ii) ZP4 ZNPs and (iii) ZP4-ANG2 ZNPs

Figure 3A highlights the physicochemical properties of the different ZNP nanoformulations. The PEGylation of the ZNPs (ZP4 ZNPs) caused a decrease in the hydrodynamic diameter of the nanoparticles compared with the pure zein nanoformulation (NF ZNPs; approximately 140 nm *versus* 80 nm). This can possibly be due to the amphiphilic nature of PEG, which led to it acting as a surfactant reducing the surface tension of the particles, changing its physical properties and therefore, decreasing its hydrodynamic diameter [33]. This decrease in hydrodynamic diameter improved the nanoformulation, considering that an average size smaller than 100 nm is generally more suitable for the purpose of BBB crossing [9]. Regarding PDI, all nanoformulations presented values up to around 0.3, which is generally considered to indicate a relatively monodisperse or narrowly distributed particle size population, suggesting uniform size distribution. The PEGylated ZP4 ZNPs presented a decrease in the zeta-potential to near neutrality compared with the pure zein NF ZNPs nanoformulation (approximately 20 mV *versus* 3 mV). This has been rendered useful to increase the circulation time of the nanoparticles [34], and to possibly lead to improved penetration through the BBB, like in the case of GBM [35]. A possible reason for this is due to the stealth effect of the PEG on decreasing the electrostatic potential and shifting the position of the shear plane outwards from the particle surface [36]. The positive zeta-potential of NF ZNPs was attributed to the protonation of the -NH_2_ groups of zein (isoelectric point around 6.2) upon dilution, which would have taken place at the dispersant’s pH of 4.5 [37].

TEM analysis depicted spherical-shaped particles with consistently smooth surfaces across all nanoparticle formulations, indicating uniformity in morphology. Additionally, the size distribution was found to be identical for all nanoparticle formulations, as illustrated in Fig. 3B.

AE and DL values were determined by HPLC analysis, and presented an increase for the PEGylated nanoformulations (average of 50–70% AE and 4–6% DL) in comparison to the non-PEGylated ZNPs (average of 25% AE and 2% DL) (Table 4). This increase is in concordance to the decrease of hydrodynamic diameter of the NPs composed by PEGylated zein. This could primarily be because smaller nanoparticles offer a larger surface area-to-volume ratio [38], allowing for more efficient interaction and entrapment of the encapsulated drug.

**Table 4:** Summary of AE and DL values for the different nanoformulations of zein loaded with DTX. Data presented as mean ± STD (*n* = 3, each *n* corresponding to a different nanoformulation batch)

Formulation code	AE (%)	DL (%)
*NF ZNPs*	25.02 ± 9.48	2.16 ± 0.82
*ZP4 ZNPs*	60.30 ± 13.3	5.00 ± 1.06
*ZP4-ANG2 5% ZNPs*	68.99 ± 13.1	5.69 ± 1.02
*ZP4-ANG2 10% ZNPs*	48.48 ± 2.27	4.04 ± 0.19
*ZP4-ANG2 20% ZNPs*	45.38 ± 2.36	3.78 ± 0.20

### In vitro cytotoxicity in glioblastoma cells

After demonstrating successful PEGylation and functionalization with ANG2 of zein, and the ability of the chemical conjugate to form nanoparticles of relatively uniform size around 100 nm with a high DTX loading value, it was necessary to prove that the encapsulated drug retains its anti-tumor activity. For this purpose, we utilized GBM cells as a cellular model for efficacy testing, although it is expected that our brain targeting nanosystem will enable the encapsulation of a variety of drugs with different therapeutic targets within the scope of brain diseases.

**Fig. 4:**
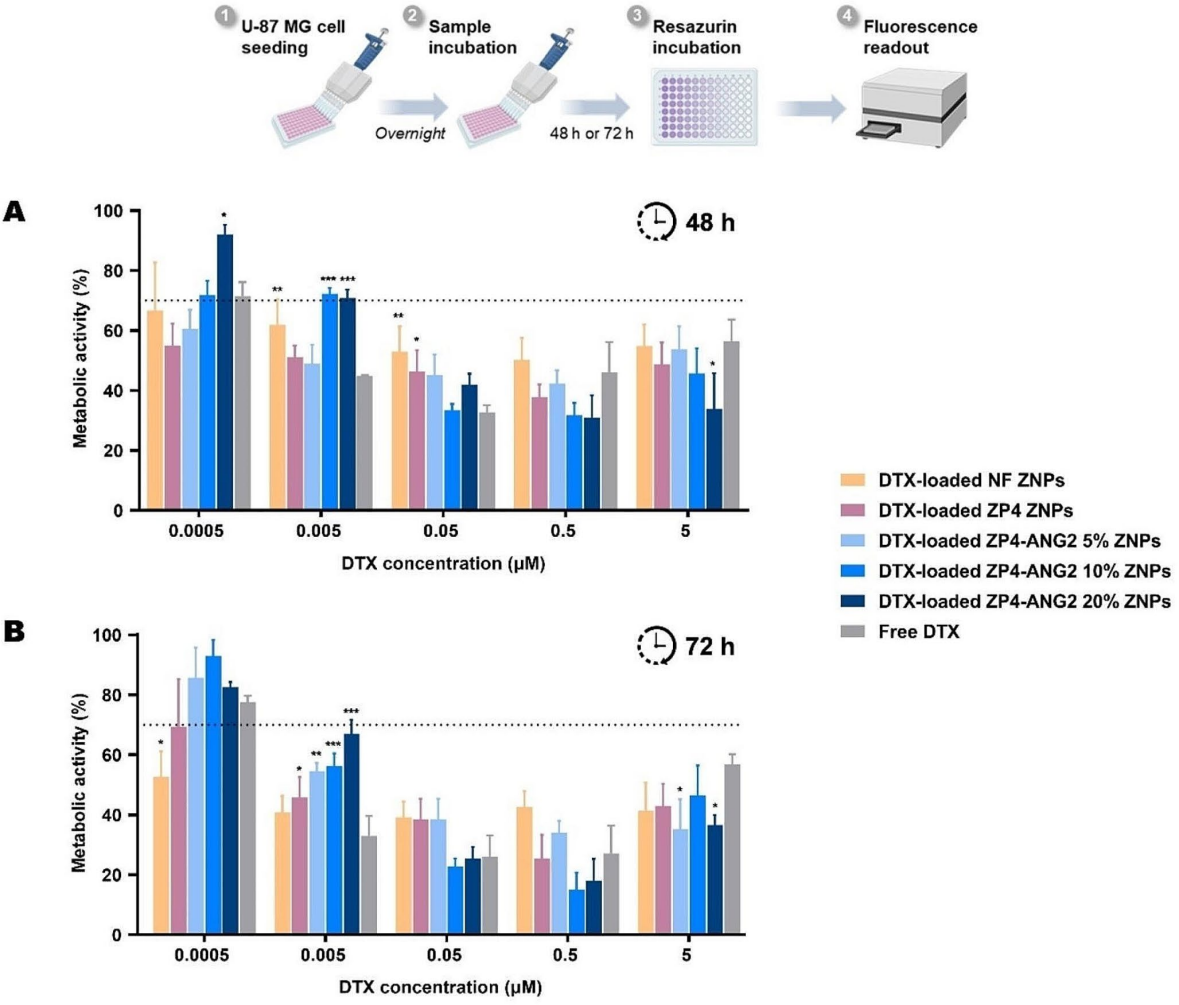
Cytotoxicity of the different DTX-loaded ZNP nanoformulations and free DTX control against U-87 MG GBM cells after **(A)** 48 h and **(B)** 72 h incubation (concentrations related to loaded DTX). Data presented as mean ± STD (*n* = 3, each *n* corresponding to a different nanoformulation batch). All comparisons were performed using two-way ANOVA followed by the Dunnett’s test (*p < 0.05, **p < 0.01, or ***p < 0.001 relative to the free DTX group)

The cytotoxicity of ZNPs was tested against the U-87 MG GBM cell line over 48 h and 72 h (Fig. 4), using a DTX concentration ranging from 0.0005 µM to 5 µM. Owing to the mechanism of action of DTX of arresting the cell cycle, which has a timespan exceeding 24 h [39], the studies were performed for longer time periods.

Increasing the concentration of DTX loaded into ZNPs beyond 0.0005 µM incurred significant reduction of metabolic activity on GBM cells, where a reduction below the threshold of 70% [40] was seen after both 48 h and 72 h. This cytotoxicity was evident upon direct treatment with pure zein, PEGylated zein, as well as PEGylated and ANG2 functionalized ZNPs (5%, 10% and 20% ANG2), comparable to the profile of free DTX at the same used concentrations. This drug-associated cytotoxicity strongly suggests the maintenance of the anti-tumor activity of DTX upon loading into ZNPs.

To further confirm that the imposed metabolic activity impairment was a result of the treatment with DTX rather than the nanoparticle matrix itself, unloaded pure zein, PEGylated zein, as well as PEGylated and ANG2 functionalized ZNPs were tested against U-87 MG GBM cells for 72 h at the same concentrations used for the loaded nanoformulations (Figure S2). Data have shown no significant impairment in metabolic activity for all formulations up to a concentration equivalent to 0.5 µM of loaded DTX, therefore confirming the safety of their matrix.

In general, the metabolic activity of GBM cells decreased in a dose-dependent manner after treatment, and lower levels were reached at 72 h compared to 48 h. This could possibly be due to mechanism of action of DTX of arresting the cell cycle, which normally takes around 72 h [41]. PEGylated and ANG2 functionalized ZNPs caused a similar impact on metabolic activity compared to pure zein and PEGylated zein ZNPs. Moreover, PEGylated and ANG2 functionalized ZNPs demonstrated a similar or slightly lower decrease on cell metabolic activity compared to the non-nanoparticulate drug control, free DTX. This was anticipated since free drug molecules are immediately available to easily interact with the cell layers, thereby exerting a faster anti-proliferative effect. Whereas, for the nanoparticulate samples, including PEGylated and ANG2 functionalized ZNPs, drug molecules become only available upon DTX diffusion across the zein matrix and, in a later stage, matrix erosion.

### In vitro permeability across the BBB

The primary goal of chemically modifying the zein matrix with ANG2, a pioneering approach, was to facilitate brain-targeted drug delivery, which has remained one of the major challenges in pharmaceutical technology.

**Fig. 5:**
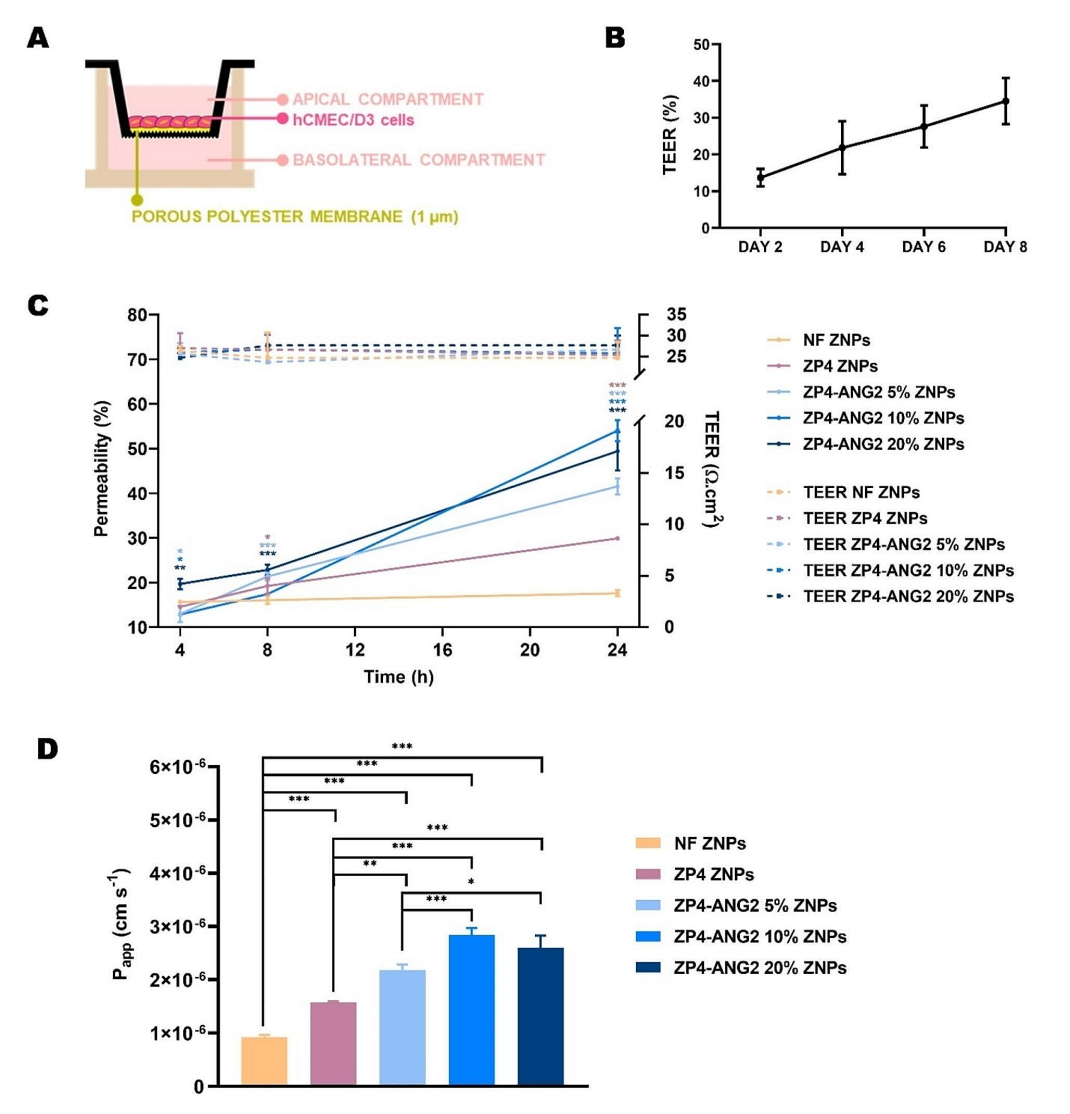
Blood-to-brain in vitro permeability study. **(A)** Scheme of the hCMEC/D3 BBB in vitro model. **(B)** Monitorization of TEER for the hCMEC/D3 BBB in vitro model from day 2 to day 8. **(C)** Cumulative permeability percentage and TEER values of the model after incubation with the different nanoformulations for 4 h, 8 h and 24 h. Statistical comparison relative to the NF ZNPs group. **(D)** Calculated P_app_ for the 24 h time-point. Data presented as mean ± STD (*n* = 3, each *n* corresponding to a different nanoformulation batch). All comparisons were performed using two-way ANOVA followed by the Dunnett’s test (*p < 0.05, **p < 0.01, or ***p < 0.001)

Studies in a hCMEC/D3 BBB in vitro model (Fig. 5A) were performed to assess the blood-to-brain permeability of the different ZNP nanoformulations, thus allowing us to extrapolate their ability to reach the targeted site of action, namely the brain parenchyma.

ZNP nanoformulations were labeled with C6, which was used as a drug surrogate, as extensively reported in literature [22]. Different concentrations of C6 analogous with pre-performed permeability studies were tested [17, 21]. A concentration of 600 µg/mL of C6 labeled nanoparticles was chosen after confirmation of lack of significant 24 h cytotoxicity in hCMEC/D3 BBB endothelial cells, as confirmed through resazurin assays (Figure S3). The 24 h timepoint was anointed as the final timepoint of the study. Pre-incubation TEER values of the hCMEC/D3 cell monolayer ranged from 5 to 35 $\text{?.}\text{cm}\text{2}$, being consistent with TEER values pre-established for this specific in vitro model (Fig. 5B) [24]. An increase of TEER values against the blank was observed up till the day of treatment (day 8), indicating the maturation of the endothelial cells’ tight junctions [42] and the formation of a monolayer.

At 4 h and 8 h, no biologically relevant differences in percentage of permeability were found between all testing conditions. However, at 24 h, significant differences were found in percentage of permeability values, namely ZP4-ANG2 10% ZNPs > ZP4-ANG2 20% ZNPs > ZP4-ANG2 5% ZNPs > ZP4 ZNPs > NF ZNPs. In contrast to previous studies indicating that PEGylation reduces cellular interactions [34], it was deduced that PEGylated ZNPs (both non-functionalized ZP4 ZNPs and ANG2 functionalized ZP4-ANG2 ZNPs) exhibited enhanced permeation characteristics at the 24 h timepoint when compared to non-PEGylated ZNPs (NF ZNPs; approximately 3-times increase in percentage of permeability (Fig. 5C) and P_app_ (Fig. 5D)). This could possibly be due to the stability enhancing effects of PEG in biologically mimetic environments [43], consequently improving the ZNPs’ likelihood to traverse the BBB monolayer intact. Alternatively, this enhanced permeability could be attributed to the slightly smaller hydrodynamic diameter of the PEGylated ZNPs [44], which accelerates their cellular uptake and transcellular permeability.

Remarkably, ZP4-ANG2 ZNPs exhibited significantly higher blood-to-brain permeability across the BBB in vitro model compared to non-PEGylated NF ZNPs (around 3-times increase in percentage of permeability for ZP4-ANG2 5% and ZP4-ANG2 20% ZNPs, and 4-times increase for ZP4-ANG2 10% ZNPs (Fig. 5C)) and PEGylated ZP4 ZNPs (around 1.5-times increase in percentage of permeability for ZP4-ANG2 5% ZNPs, and 2-times increase for ZP4-ANG2 10% and ZP4-ANG2 20% ZNPs (Fig. 5C)), suggesting the key role of receptor-mediated transcytosis via ANG2 binding to LRP-1. Increasing the percentage of ANG2 in the nanoformulation from 5 to 10% and 20% significantly improved percentage of permeability (Fig. 5C) and P_app_ (Fig. 5D). Nonetheless, the increase in ANG2 content in the final nanoformulation from 10 to 20% yielded no significant change in in vitro blood-to-brain permeability, as shown by permeability percentage values (Fig. 5C), suggesting the possibility of receptor saturation [45]. The most substantial difference in permeability between formulations was observed at the 24 h timepoint, thereof denoting that the influence of ANG2 becomes more pronounced past the 8 h timepoint.

Throughout the permeability assay, TEER values showed no significant fluctuations across all nanoformulations (Fig. 5C). This consistency indicates that the integrity and intactness of the tight junction-held BBB in vitro monolayer were preserved throughout the entire experiment. If there had been any disruption of the monolayer, we would have observed a similar increase in permeability levels across all nanoformulations, which did not occur.

These results clearly demonstrated the ability of ANG2 functionalization to ameliorate the accumulation of the nanoformulations into the brain. With the use of a PEGylated and ANG2 functionalized zein matrix, the in vitro BBB permeability of the nanoformulation was efficiently maximized, with an overall increase of up to 4 times. This demonstrates an enhanced capacity of traversing the BBB, with potential to increase the efficacy of delivering therapeutics to the brain.

## Conclusions and future perspectives

In conclusion, we effectively managed, for the first time, to covalently functionalize zein protein with PEG and ANG2 prior to ZNPs manufacture. Physicochemical characterization tests confirmed the formation of 100 nm particles with mostly monodisperse populations, with a DTX DL of up to 6%. Additionally, we demonstrated the cytotoxicity of DTX-loaded ZNPs against the U-87 MG GBM cell line parallel to the direct use of DTX in its native form, which confirmed the ability of our ZNPs to sustain the cytotoxic potential of the loaded chemotherapeutic. Moreover, we were able to showcase the upgraded in vitro blood-to-brain permeability resulting from the PEGylation of the ZNPs, which was exceeded upon PEG functionalization with ANG2, proving the enhanced effect and crucial involvement of LDLR in the brain delivery of the ZNPs. Saturation of LDLR was demonstrated upon increasing the concentration of ANG2 in the nanoformulation from 10 to 20%, indicating the sufficiency of using 10% ANG2 for the targeting. With the ANG2-dependent enhancement in permeability across a BBB in vitro model, and the evident cytotoxicity of the DTX-loaded ZNPs against tumour cells, the developed nanosystem offers an innovative approach for future treatment of GBM. For future investigations, we propose complementing the in vitro data by conducting experiments using 3D BBB-GBM interplay cell models. Lastly, in vivo studies are also crucial to validate the findings in a relevant disease model. In summary, our brain-targeted zein nanosystem ensures reproducible production, mitigating the risk of premature drug release common in post-functionalization. This innovative approach also holds promise for addressing various neurological disorders necessitating BBB passage, potentially benefiting other disease conditions.

## Electronic supplementary material

Below is the link to the electronic supplementary material.

Supplementary Material 1

## Data Availability

No datasets were generated or analysed during the current study.
