# Supplementary material for: Chemical engineering of zein with polyethylene glycol and Angiopep-2 to manufacture a brain-targeted docetaxel nanomedicine for glioblastoma treatment
Source: Drug Deliv Transl Res. 2024 Jul 15;14(12):3585–98. doi: 10.1007/s13346-024-01659-x (PMC11499337; doi:10.1007/s13346-024-01659-x)
Supplement: Supplementary file 1 — Supplementary Material 1 [file 13346_2024_1659_MOESM1_ESM.docx]

Supporting Information

Chemical engineering of zein with polyethylene glycol and Angiopep-2 to manufacture a brain-targeted docetaxel nanomedicine for glioblastoma treatment

*Seem Awad, Marco Araújo, Paulo Faria, Bruno Sarmento*, Cláudia Martins**



Figure S1. Bromocresol staining to qualitatively follow the disappearance of the carboxylic acid functionality.


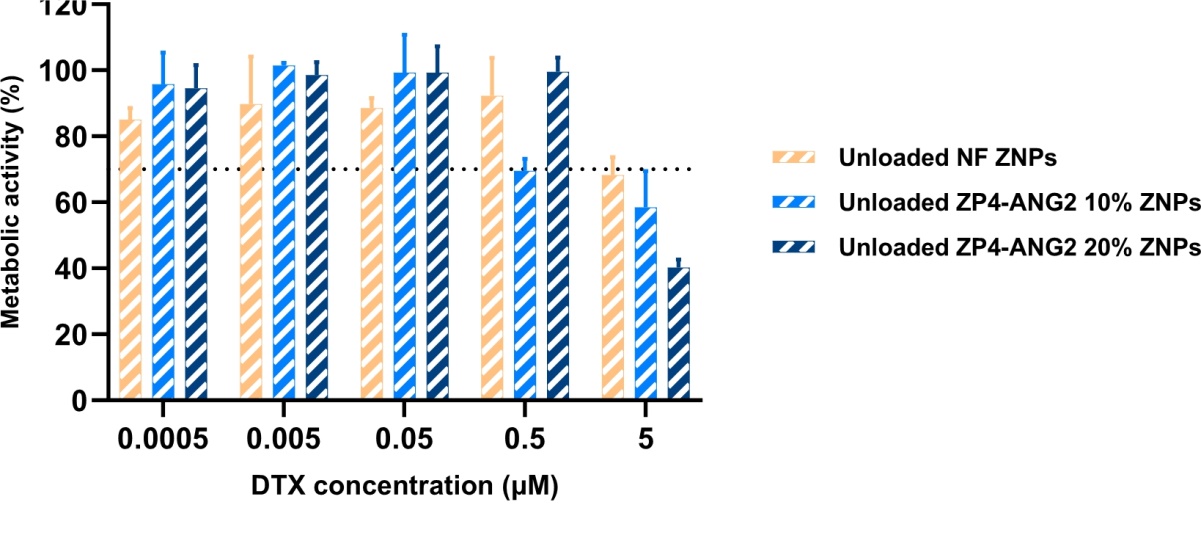


Figure S2. U-87 MG GBM cell metabolic activity after exposure to different concentrations of unloaded ZNP nanoformulations for 72 h (concentrations related to equivalent loaded DTX). Data presented as mean ± STD (*n* = 3, each *n* corresponding to a different nanoformulation batch).


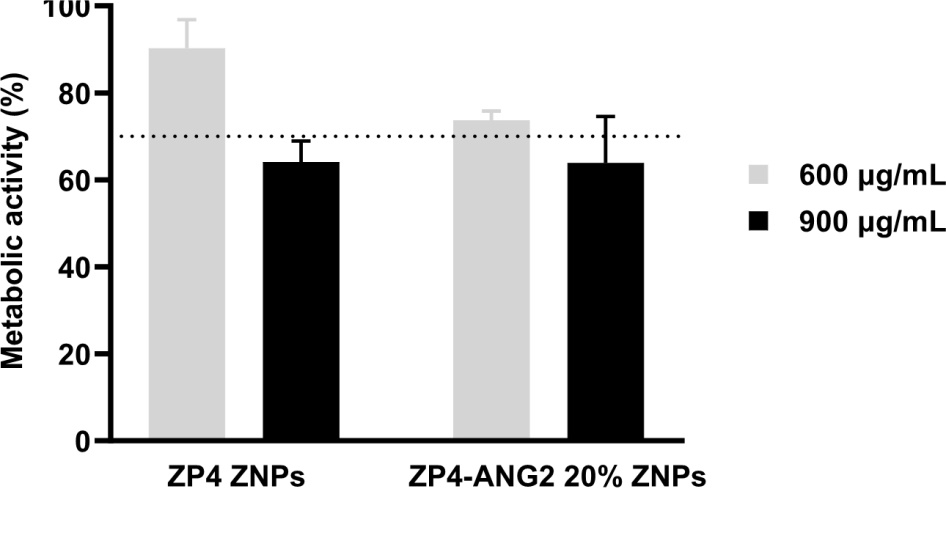


Figure S3. hCMEC/D3 cells metabolic activity after exposure to different concentrations of C6-loaded ZP4 and ZP4-ANG2 20% ZNPs for 24 h. This study aimed at determining the maximum tolerated concentration for *in vitro* permeability experiments. Data presented as mean ± STD (*n* = 3, each *n* corresponding to a different batch).
